# Rosuvastatin suppresses TNF-α-induced matrix catabolism, pyroptosis and senescence via the HMGB1/NF-κB signaling pathway in nucleus pulposus cells: Role of rosuvastatin in alleviating intervertebral disc degeneration

**DOI:** 10.3724/abbs.2023026

**Published:** 2023-05-24

**Authors:** Weijian Chen, Zhihuai Deng, Jianxiong Zhu, Liang Yuan, Shuangxing Li, Yangyang Zhang, Jiajun Wu, Zhengqi Huang, Tianyu Qin, Wei Ye

**Affiliations:** 1 Department of Orthopedics the Fifth Affiliated Hospital of Guangzhou Medical University Guangzhou 510705 China; 2 Guangdong Provincial Key Laboratory of Malignant Tumor Epigenetics and Gene Regulation Medical Research Center Sun Yat-sen Memorial Hospital of Sun Yat-sen University Guangzhou 510120 China; 3 Department of Spine Surgery Sun Yat-sen Memorial Hospital of Sun Yat-sen University Guangzhou 510120 China; 4 Department of Orthopedics the Eighth Affiliated Hospital of Sun Yat-sen University Shenzhen 518033 China

**Keywords:** intervertebral disc degeneration, rosuvastatin, high-mobility group box 1, NF-κB signaling pathway

## Abstract

Intervertebral disc degeneration is mainly caused by irregular matrix metabolism in nucleus pulposus cells and involves inflammatory factors such as TNF-α. Rosuvastatin, which is widely used in the clinic to reduce cholesterol levels, exerts anti-inflammatory effects, but whether rosuvastatin participates in IDD remains unclear. The current study aims to investigate the regulatory effect of rosuvastatin on IDD and the potential mechanism. *In vitro* experiments demonstrate that rosuvastatin promotes matrix anabolism and suppresses catabolism in response to TNF-α stimulation. In addition, rosuvastatin inhibits cell pyroptosis and senescence induced by TNF-α. These results demonstrate the therapeutic effect of rosuvastatin on IDD. We further find that HMGB1, a gene closely related to cholesterol metabolism and the inflammatory response, is upregulated in response to TNF-α stimulation. HMGB1 inhibition or knockdown successfully alleviates TNF-α-induced ECM degradation, senescence and pyroptosis. Subsequently, we find that HMGB1 is regulated by rosuvastatin and that its overexpression abrogates the protective effect of rosuvastatin. We then verify that the NF-κB pathway is the underlying pathway regulated by rosuvastatin and HMGB1. *In vivo* experiments also reveal that rosuvastatin inhibits IDD progression by alleviating pyroptosis and senescence and downregulating HMGB1 and p65. This study might provide new insight into therapeutic strategies for IDD.

## Introduction

Low back pain (LBP) is considered one of the leading causes of disability worldwide and causes great social and economic burdens [1]. Intervertebral discs (IVDs) are pads of fibrocartilage lying between adjacent vertebraes that contain the nucleus pulposus (NP), surrounding annulus fibrosus and upper and lower cartilage endplates [2]. Intervertebral disc degeneration (IDD) is one of the main causes of LBP [3]. Various factors contribute to the occurrence of IDD, including mechanical overload, aging, and genetic susceptibility [ 4– 6] . As IDD progresses, nucleus pulposus cells exhibit a series of changes, such as senescence, cell death and phenotypic changes, resulting in the secretion of inflammatory cytokines, such as tumor necrosis factor α (TNF-α) and interleukin 1β (IL-1β) [ 7, 8] . Consequently, the homeostasis of extracellular matrix (ECM) metabolism is disturbed: the anabolism of ECM components, including type II collagen (COL2A) and aggrecan (ACAN), is suppressed, whereas the catabolism of ECM components, such as matrix metalloproteinases (MMPs) and a disintegrin-like and metalloprotease with thrombospondin type-1 motif (ADAMTSs), is promoted [9]. Moreover, the impact of inflammatory cytokines not only disturbs ECM metabolism but also promotes senescence and cell death, which in turn accelerates IDD [10].

Pyroptosis is a type of programmed cell death triggered by inflammatory cytokines such as TNF-α and IL-1β [11]. The classic mechanism of pyroptosis involves the assembly and priming of inflammasomes such as NLRP3 inflammasomes, followed by the activation of caspase-1 and gasdermin D (GSDMD). Pores are then formed in the cell membrane, and proinflammatory cytokines such as IL-1β and interleukin 18 (IL-18) are released, which ultimately causes cell death and a cascade of inflammatory responses [12]. Our previous study found that cholesterol could induce pyroptosis in NP cells [13]. In addition, accumulating evidence proves that pyroptosis is involved in IDD [14]. Therefore, targeting the process of pyroptosis may be a therapeutic strategy for IDD.

Cell senescence represents the arrest of the cell cycle and is considered a hallmark of aging. The induction of senescence involves a constitutive DNA damage response and the release of inflammatory cytokines [15], resulting in the activation of senescence programming governed by the p53‒p21‒pRB pathway and p16‒pRB pathway [16]. Senescence decreases the number of functional cells in intervertebral discs, and senescent NP cells aggravate IDD progression via paracrine signaling to induce the senescence of adjacent cells [17].

Statins are a group of 3-hydroxy-3-methyl glutaryl coenzyme A (HMG-CoA) reductase inhibitors [18]. In addition to their clinical use in reducing cholesterol levels, they also exert important anti-inflammatory and antioxidative effects in the context of degenerative diseases such as osteoarthritis and Alzheimer’s disease [ 19– 21] . Our previous study revealed that atorvastatin promoted autophagy and decreased NLRP3 activity in TNF-α-treated NP cells [22]. Rosuvastatin is a fully synthetic statin. Compared with other molecules of the same class, such as atorvastatin, rosuvastatin exerts higher efficacy in reducing cholesterol levels while causing fewer adverse effects [ 23– 25] . In addition to its benefits in cholesterol regulation, this statin is superior to atorvastatin in anti-inflammatory and cardiovascular system protection [ 26, 27] . Considering the role of inflammation in IDD progression and the efficacy of rosuvastatin against IDD, exploring the effect of rosuvastatin on IDD may provide insight into IDD treatment.

High-mobility group box 1 (HMGB1) is a highly conserved protein that is located in the nucleus in eukaryotes [28] and acts as a proinflammatory factor after being activated by inflammatory stimuli, hypoxia and oxidative stress [29]. Studies have shown that targeting HMGB1 could alleviate IDD and osteoarthritis progression [ 27, 30] . In addition, HMGB1 is related to cholesterol metabolism. Aberrant cholesterol metabolism promotes the release of HMGB1 by hepatocytes, resulting in nonalcoholic steatohepatitis [31]. Considering the function of HMGB1 in cholesterol metabolism and IDD, whether rosuvastatin can alleviate IDD by targeting HMGB1 is worthy of discussion.

In this study, we investigated whether rosuvastatin could reverse TNF-α-induced degeneration in rat NP cells. Our data revealed that rosuvastatin protected NP cells from the inflammatory response by regulating the HMGB1/NF-κB pathway, and this finding suggested a potential therapeutic effect of rosuvastatin on IDD.

## Materials and Methods

### Animals and groups

The animal experiment procedures were approved by the Animal Care and Use Committee of Sun Yat-sen University (No. SYSU-IACUC-2022-000067) (Guangzhou, China). In the *in vivo* experiment, 12 male Sprague‒Dawley rats (aged 10 weeks) were randomly divided into 3 groups: the control group, IDD group, and the rosuvastatin group. After anesthetization via an intraperitoneal injection of 3% pentobarbital sodium (30 mg/kg), the Co7-8 IVD was punctured with a 20-G needle to establish the experimental model of IDD. All needles were rotated 360° and held in place for 30 s [32]. After the operation, rosuvastatin (10 mg/kg/d; GlpBio, Montclair, USA) [33] was dissolved in saline and intraperitoneally injected, whereas the control group and the IDD group received the same amount of saline. Daily injections started on the day of the operation and were administered for 4 weeks.

### Isolation and culture of rat NP cells

NP cells were derived from normal Sprague-Dawley rat IVDs. After euthanasia, the rat IVDs were collected, cut into pieces, placed in a 1.5-mL EP tube containing 10% FBS in DMEM (Gibco, New York, USA) and subsequently washed three times with phosphate-buffered saline (PBS). The tissue was then digested with type II collagenase at 37°C with 5% CO _2_ for 2 h and washed three times with 10% FBS (Gibco) in DMEM. After digestion, the resuspended NP cells were cultured in medium supplemented with 10% FBS at 37°C with 5% CO _2_, and the culture medium was changed every 3 days. Cells were passaged when they reached 80% confluence. Cells in passages 3–5 were used for the experiments.

### Cell transfection

The HMGB1 plasmid was constructed by Gene Create (Wuhan, China). An siRNA against HMGB1 (siHMGB1) with a target sequence of 5′-GAGUCCUGGAUGAUACUAATT-3′ and a simulated sequence of 5′-UUCUCCGAACGUGUCACGUTT-3′ was constructed by GenePharma (Shanghai, China). NP cells were transfected using Lipofectamine 3000 (Invitrogen, Carlsbad, USA). Briefly, NP cells were seeded in 6-well plates. After reaching 60%–70% confluence, plasmid (2 μg) or siRNA (150 pmole) was added along with Lipofectamine reagent and Opti-MEM (Gibco). Twelve hours after transfection, the medium was replaced by fresh medium, and regular culture was performed for certain experiments.

### Quantitative real-time polymerase chain reaction (qRT-PCR)

According to the manufacturer’s instructions, total RNA was extracted from NP cells using TRIzol reagent (Takara, Dalian, China). qRT-PCR was performed using cDNA, forward and reverse primers and enzyme-free water with a Roche Light Cycler 96 (Roche Diagnostic, Mannheim, Germany). The primers were synthesized by Generay (Shanghai, China), and the primer sequences for qRT-PCR are listed in Table 1. The 2 ^–ΔΔCt^ method was used for calculation, and mRNA expression of each target gene was normalized to that of *β-actin*.

**Table:** **Table 1** Sequences of primers used for qRT-PCR

Gene	Forward primer (5′→3′)	Reverse primer (5′→3′)
*β-Actin*	GAGAGGGAAATCGTGCGT	GGAGGAAGAGGATGCGG
*ACAN*	ACACGGCTCCACTTGATTCTT	CTTGGTCTTTGTGACTCTTGCG
*COL2A*	CACGCTCAAGTCGCTGAA	AGCCCTGGTTGGGATCA
*MMP3*	ACCTATTCCTGGTTGCTG	GGTCTGTGGAGGACTTGTA
*ADAMTS5*	AAAACTGGCGAGTACCTT	TCCTTTGTGGCTGAATAG
*p16*	CCGAGAGGAAGGCGAAC	GGGGTACGACCGAAAGTG
*p21*	CACTGGACTATGAGGACTGGA	TGAGCTTGAGAACTGGTGTG
*HMGB1*	ATGCCTTTGCCCTTCTATC	ATCCGCTTTCCTTGTATCTG

### Western blot analysis

NP cells were lysed on ice in a mixture of RIPA lysis buffer with protease and phosphatase inhibitors for 20 min to extract total proteins, and the protein concentrations were determined with a BCA protein assay kit (CWBIO, Beijing, China). The proteins were separated on sodium dodecyl sulfate-polyacrylamide gels and then transferred to PVDF membranes. After being blocked with 5% BSA at room temperature for 1 h, the membranes were incubated with the corresponding primary antibodies at 4°C overnight. The antibodies were as follows: anti-β-actin (1:1000; ABclonal, Wuhan, China), anti-p-p65 (1:1000; ABclonal), anti-p65 (1:1000; ABclonal), and anti-HMGB1 (1:1000; ABclonal), anti-ADAMTS5 (1:1000; Abcam, Cambridge, UK), anti-ACAN (1:1000; Abcam), anti-COL2A (1:1000; Abcam), anti-p21 (1:1000; Abcam), anti-p16 (1:1000; Abcam), anti-MMP3 (1:1000; Cell Signaling Technology, Beverly, USA), anti-NLRP3 (1:1000; Cell Signaling Technology), anti-GSDMD-NT (1:1000; Cell Signaling Technology), anti-cleaved caspase-1/p20 (1:1000; Affinity, Melbourne, Australia), and anti-GSDMD-NT (1:1000; Huabio, Hangzhou, China). After primary antibody incubation, the membranes were subjected to three 10-min washes with Tris-buffered saline plus Tween (TBST; 10 mM Tris-HCl, pH 8.0, 150 mM NaCl and 0.1% Tween-20), followed by incubation with HRP-conjugated secondary antibodies (1:5000; Abclonal) at room temperature for 1 h. Finally, the bands were detected with a hypersensitive luminescence solution on a scanning system (Syngene G:BOX ChemiXT4; Syngene, Cambridge, UK and BLT GelView 6000 Pro; Biolight Biotechnology, Guangzhou, China).

### Transmission electron microscopy (TEM)

The pretreated cells were digested with trypsin and centrifuged at 1000 *g* for 5 min in a 1.5-mL microcentrifuge tube, and the cell pellet was visible to the naked eye. The pellet was fixed with 2.5% glutaraldehyde overnight at 4°C, and after being washed three times with PBS, the pellets were postfixed again in 1% osmium tetroxide for 2 h at 4°C. The NP cells were then dehydrated with graded concentrations of alcohol (50%, 70%, 80%, and 90%), washed three times with PBS and dehydrated with acetone. After being soaked in 3:1 100% acetone:embedding solution and 1:1 100% acetone:embedding solution, the cells were incubated overnight in pure embedding solution at 4°C. The samples were then cut into ultrathin slices with a thickness of approximately 100 nm using a Leica UC7 ultramicrotome and subjected to electron staining with uranium dioxy acetate and lead citrate. Finally, the samples were observed and photographed under a Tecnai G2 Spirit transmission electron microscope (FEI Company, Hillsboro, USA).

### β-Galactosidase assay

The cells were plated in 6-well plates at approximately 30% confluence. After pretreatment, the medium was removed, and the cells were washed once with PBS. Then, 1 mL of β-galactosidase staining fixative was added, and the cells were fixed at room temperature for 15 min. The cells were then washed three times with PBS, the mixture was added according to the instructions of the β-galactosidase staining kit (Beyotime, Shanghai, China), and the cells were incubated in the absence of carbon dioxide overnight at 37°C. Finally, images were obtained under an inverted fluorescence microscope (Olympus, Tokyo, Japan).

### Immunofluorescence analysis

After the indicated treatment, the medium was removed, and the cells were washed three times with PBS and fixed with 4% paraformaldehyde (PFA) at room temperature for 20 min. The cells were then permeabilized with 0.3%–0.5% Triton X-100 (Solarbio, Beijing, China) for 10 min and incubated with 10% goat serum blocking solution for 1 h. Primary antibodies, including anti-p65 antibody (1:100; ABclonal) and anti-COL2A antibody (1:100; Abcam), were directly added, and the cells were then incubated overnight at 4°C. After 3 times wash with PBS, the samples were incubated with conjugated AffiniPure goat anti-rabbit IgG (FITC or Cy3, 1:100; BOSTER Biological Technology, Wuhan, China) in the dark for 1 h. Subsequently, the cell nuclei were stained with DAPI (Solarbio, Beijing, China) at room temperature in the dark for 5 min and immediately observed under an inverted fluorescence microscope.

### Cell counting kit-8 (CCK-8) assay

Rat NP cells were digested, and 100 μL cell suspension (approximately 5000 cells) was inoculated in each well of a 96-well cell culture plate and then incubated in 5% CO _2_ at 37°C. After 24 h, the original culture medium was removed from the well, and the cells were subsequently washed with PBS. Then, 100 μL of serum-free DMEM was added to each well, and the plate was incubated for 6 h for starvation. After 6 h, serum-free DMEM was substituted with 150 μL medium containing 1% serum and 1, 5, 10, 20, 25 or 30 μM rosuvastatin, and the cells were incubated at 37°C in 5% CO _2_ for 24, 48 or 72 h. At the indicated time point during stimulation, the original medium was removed and 100 μl of serum-free DMEM with 10% CCK-8 reagent was added to each well. Then, the cells were incubated at 37°C in 5% CO _2_ for 1 to 4 h in the dark. The absorbance at 450 nm was then measured with a microplate reader.

### Lactate dehydrogenase (LDH) assay

The LDH assay was performed using the CytoTox96 Non-Radioactive Cytotoxicity Assay kit (Promega, Madison, USA). Briefly, NP cells were plated in 96-well plates. After treatment, maximal LDH release was achieved by adding 10 μL of lysis solution per 100 μL of medium for 45 min. Fifty microliters of supernatant from all the wells was transferred to fresh 96-well plates and incubated for 30 min at room temperature in the dark with LDH reagent. Fifty microliters of stop solution was then added to each well and the absorbance was recorded at 492 nm.

### Immunohistochemistry assay

Rat and human IVD tissues were obtained, fixed with 4% PFA for 48 h and then decalcified and embedded in paraffin. The tissues were cut into 5-μm-thick sections that were successively dewaxed and hydrated with xylene, anhydrous ethanol and an alcohol gradient (95%, 85% and 75%). The sections were then subjected to antigen retrieval with pepsin for 20 min at 37°C, incubated with neutralized endogenous peroxidase in a humidified box for 15‒20 min and sealed with goat serum for 30 min. The slides were then incubated overnight at 4°C with primary antibodies including anti-p-p65 (1:100; ABclonal), anti-HMGB1 (1:100; ABclonal), anti-NLRP3 (1:1000; Cell Signaling Technology), anti-caspase-1/p20 (1:100; Affinity), and anti-p21 (1:100; Santa Cruz Biotech, Santa Cruz, USA). The sections were subsequently rewarmed and incubated with biotin-labeled goat anti-mouse/rabbit secondary antibodies for 20‒30 min at room temperature and HRP-conjugated streptavidin (ZSGB-BIO, Beijing, China) for 25 min. DAB (ZSGB-BIO) staining and hematoxylin staining were performed for 1 min. Finally, images were captured using an Eclipse 80i fluorescence microscope (Nikon, Tyoko, Japan).

### Hematoxylin-eosin (HE) staining and safranin O/fast green staining

The spines of the rat tail, including the IVDs and adjacent vertebrae, were separated, fixed with 4% paraformaldehyde (PFA) for 48 h, and decalcified with 10% ethylenediaminetetraacetic acid (EDTA, pH 7.5) for 30 days after being washed with PBS. All IVDs were embedded in paraffin and then cut into 5-μm midsagittal sections. The sections were dewaxed and dehydrated according to the standard procedures, and HE staining and safranin O/fast green staining (Sigma Aldrich, St Louis, USA) were then performed. Images of the sections were captured with the Eclipse 80i fluorescence microscope.

### Statistical analysis

All data are presented as the mean±standard deviation (SD) of at least three independent experiments. The experimental data were analyzed using GraphPad Prism 7 (GraphPad Software, La Jolla, USA). Student’s *t* test was used for comparisons between two groups, and multiple-group comparisons were analyzed by ANOVA. Differences were considered significant if *P*<0.05.

## Results

### Rosuvastatin restores ECM metabolism in TNF-α-treated rat NP cells

**Figure 1:**
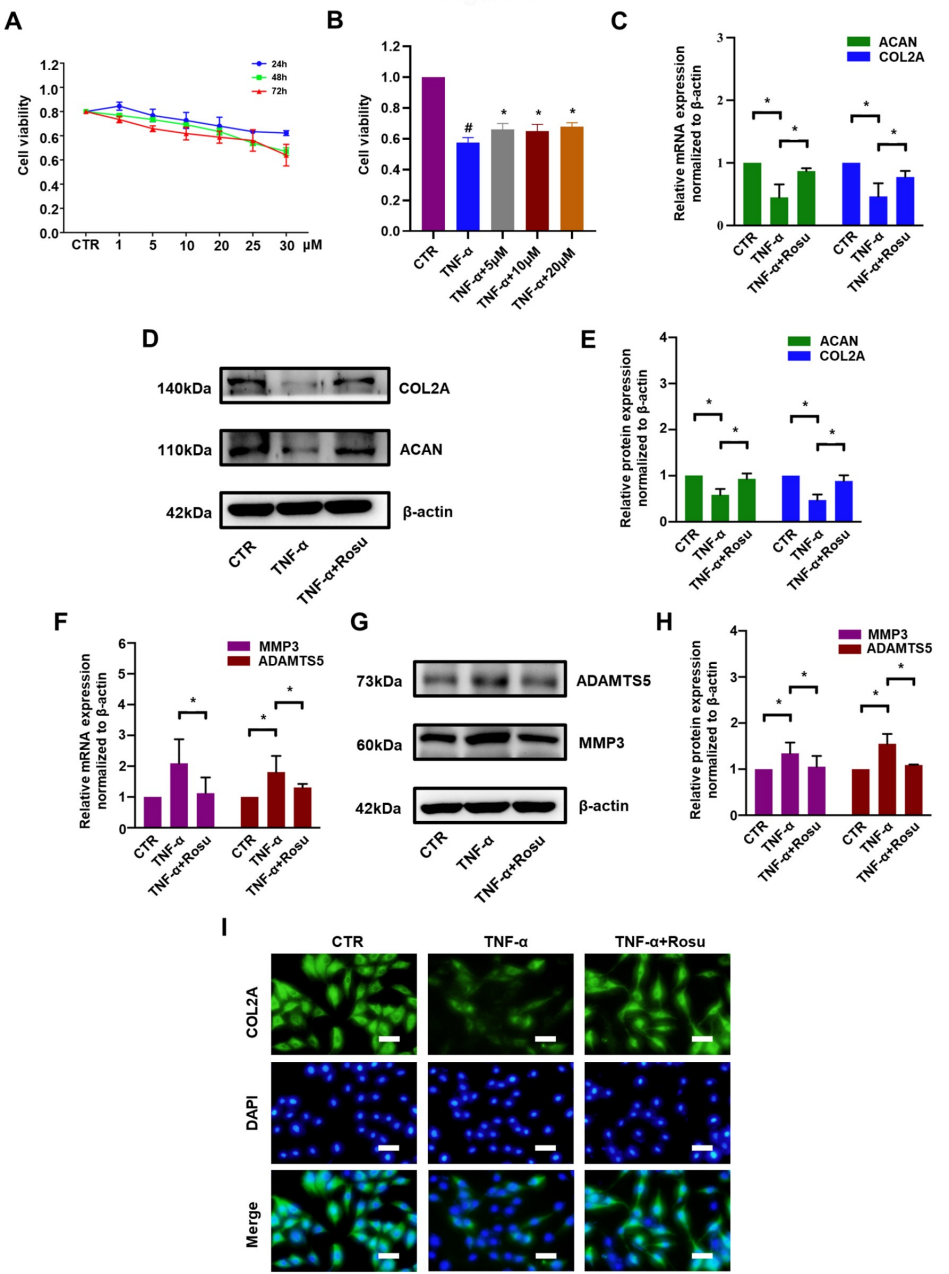
Figure 1 Rosuvastatin restored ECM metabolism in TNF-α-treated rat NP cells (A) CCK-8 assay was used to examine the toxicity of various concentrations of rosuvastatin at different time points in rat NP cells. (B) CCK-8 assay was used to examine the effect of rosuvastatin on TNF-α-treated rat NP cells for 72 h. (C) Quantitative real-time PCR was used to examine the effect of rosuvastatin on ACAN and COL2A mRNA expressions. Western blot (D) and subsequent densitometric analyses (E) were used to examine the effect of rosuvastatin on the protein expressions of ACAN and COL2A. (F) Quantitative real-time PCR was used to examine the effect of rosuvastatin on MMP3 and ADAMTS5 mRNA expressions. Western blot (G) and subsequent densitometric analyses (H) were used to examine the effect of rosuvastatin on the protein expressions of MMP3 and ADAMTS5. (I) Immunofluorescence microscopy was used to observe COL2A after treatment with rosuvastatin (scale bar: 40 μm). All experiments were repeated three times, and data are presented as the mean±SD. * P<0.05.

To determine the effect of rosuvastatin on NP cells, different concentrations of rosuvastatin (1, 5, 10, 20, 25, and 30 μM) were used to treat NP cells for different time (24, 48, and 72 h). As shown in Figure 1A, cell viability higher than 80% was obtained with rosuvastatin concentrations were lower than 25 μM at all time points. Therefore, 20 μM rosuvastatin was used in the subsequent experiments. To further investigate the effect of rosuvastatin on TNF-α-treated NP cells, different concentrations of rosuvastatin were applied along with TNF-α for 72 h. As shown in Figure 1B, the cell viability was reduced to 57% after TNF-α treatment and increased to approximately 65% with the addition of rosuvastatin. Rat NP cells were pretreated with rosuvastatin for 2 h and then treated with TNF-α (50 ng/mL) for 72 h. The mRNA level of ACAN, which was decreased by TNF-α to 45% of that in the control group, was restored to 87% by rosuvastatin, whereas the mRNA level of COL2A increased from 46% to 77% ( Figure 1C). Consistently, the ACAN and COL2A protein expression levels were decreased to approximately 50% by TNF-α, whereas rosuvastatin attenuated the decreases by almost 2-fold ( Figure 1D,E). In contrast, the mRNA levels of the ECM catabolic markers MMP3 and ADAMTS5 were significantly increased (approximately 2-fold) by TNF-α, and rosuvastatin reversed this change ( Figure 1F). Accordingly, western blot anlysis confirmed that the levels of MMP3 and ADAMTS5 were increased to 1.35-fold and 1.55-fold, respectively, by TNF-α and were reversed to 1.06-fold and 1.09-fold, respectively, when rosuvastatin was added ( Figure 1G,H). Immunofluorescence analysis was further performed to detect the effect of rosuvastatin on COL2A, and the results confirmed that the downregulation of COL2A expression induced by TNF-α was significantly reversed by rosuvastatin ( Figure 1I).

### Rosuvastatin inhibits pyroptosis and senescence induced by TNF-α in rat NP cells

**Figure 2:**
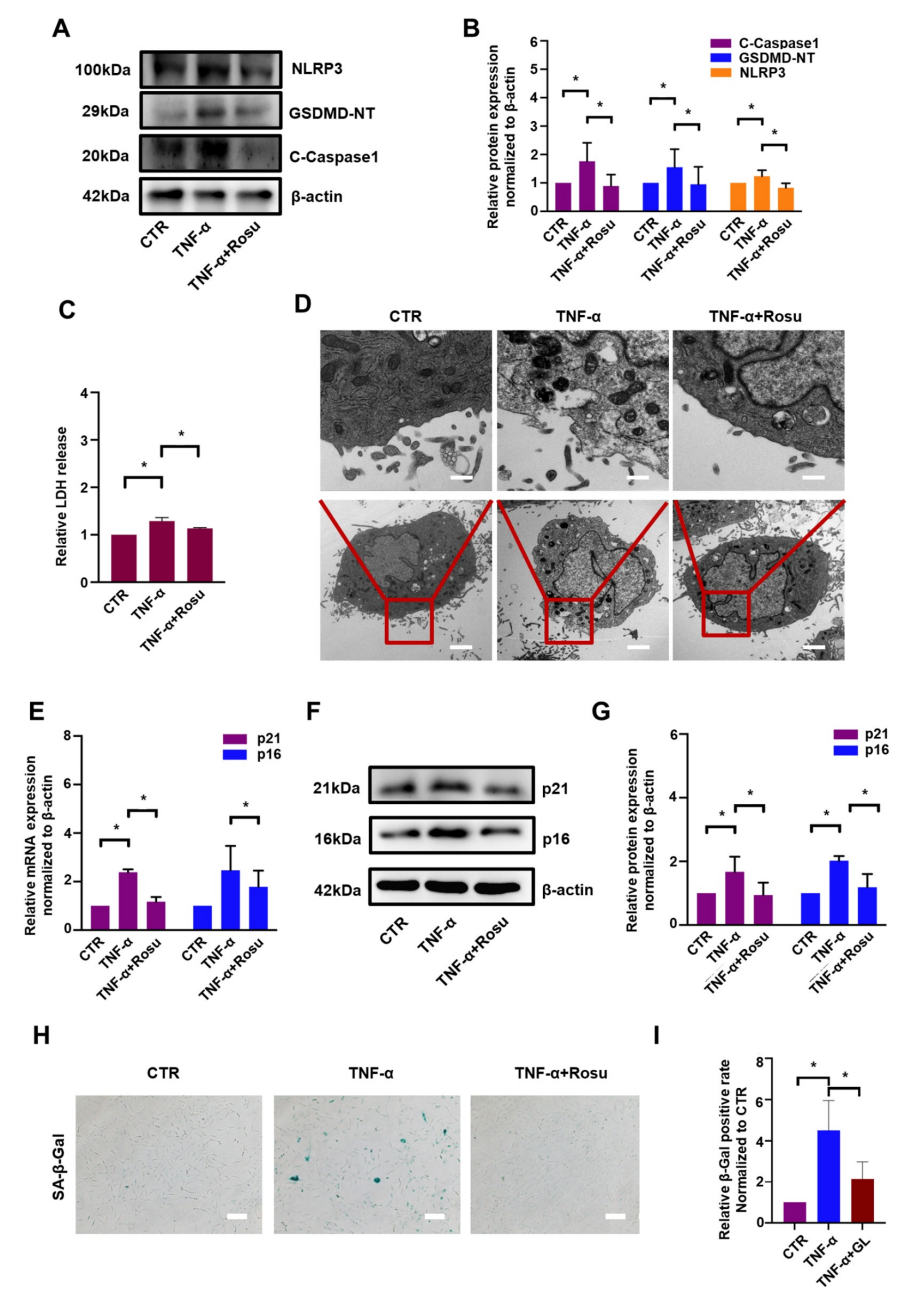
Figure 2 Rosuvastatin alleviated pyroptosis and senescence induced by TNF-α in rat NP cells Western blot (A) and subsequent densitometric analyses (B) were performed to explore the effect of rosuvastatin on pyroptosis-associated proteins. (C) LDH assay was conducted to detect membrane integrity. (D) TEM showed that rosuvastatin maintained the membrane integrity of NP cells in the presence of TNF-α (scale bar for upper panel: 500 nm, lower panel: 5 μm). (E) Quantitative real-time PCR was used to examine the effect of rosuvastatin on p16 and p21 mRNA expressions. Western blot (F) and subsequent densitometric analyses (G) were conducted to explore the effect of rosuvastatin on senescence-associated proteins. Representative micrographs (H) and quantitative analysis (I) of NP cells stained for SA-β-gal in the different treatment groups (scale bar: 10 μm). All experiments were repeated three times, and data are presented as the mean±SD. * P<0.05.

To explore whether rosuvastatin could regulate pyroptosis, western blot analysis was performed after rosuvastatin stimulation in the presence of TNF-α. The levels of NLRP3, GSDMD-NT and cleaved caspase-1 were markedly increased by TNF-α to 1.76-fold, 1.55-fold and 1.24-fold, respectively, compared to the level in the control group, but these changes were considerably reversed by rosuvastatin, with fold changes of 0.89, 0.96 and 0.83, respectively ( Figure 2A,B). To further confirm the protective effect of rosuvastatin on pyroptosis, LDH release assay was conducted to examine the permeability and integrity of the NP cell membrane. The elevation in LDH release induced by TNF-α was reversed by rosuvastatin ( Figure 2C). Furthermore, TEM was used to directly observe pyroptotic processes. As shown in Figure 2D, rosuvastatin helped maintain the membrane integrity in NP cells in the presence of TNF-α, indicating that rosuvastatin inhibits pyroptosis induced by TNF-α.

The role of rosuvastatin in TNF-α-induced NP cell senescence remains unclear. As shown in Figure 2E, compared with those in the control group, the mRNA levels of p21 and p16 were upregulated by TNF-α by 2.38-fold and 2.46-fold, respectively, but these changes were significantly reversed by rosuvastatin, with fold changes of 1.17 and 1.78, respectively. Accordingly, western blot analysis confirmed that the protein expressions of p21 and p16 were increased by approximately 2-fold in the presence of TNF-α. However, these effects were reversed by rosuvastatin, with fold changes of 0.82 and 1.25, respectively ( Figure 2F,G). Consistently, the SA-β-gal experiment revealed that the number of positive cells was significantly reduced in the rosuvastatin group in the presence of TNF-α ( Figure 2H,I).

### HMGB1 is upregulated during IDD, and HMGB1 inhibition alleviates ECM degradation in TNF-α-treated rat NP cells

**Figure 3:**
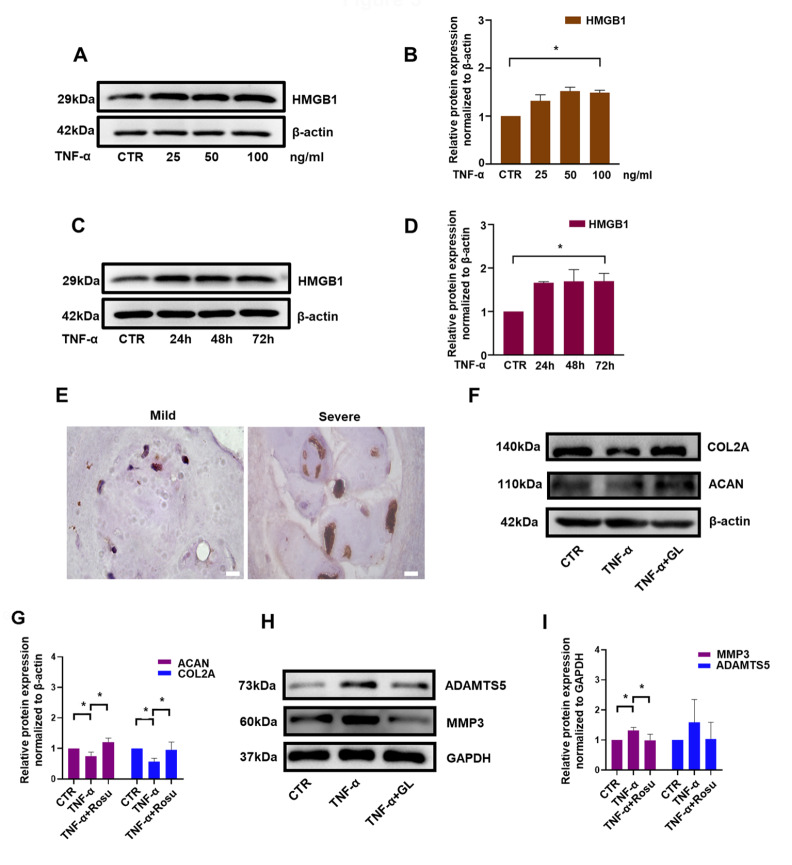
Figure 3 HMGB1 was upregulated during IDD, and HMGB1 inhibition attenuated ECM degradation in TNF-α-treated rat NP cells Western blot (A) and subsequent densitometric analyses (B) were performed to detect HMGB1 expression under treatment with different concentrations of TNF-α. Western blot (C) and subsequent densitometric analyses (D) demonstrated the expression of HMGB1 after treatment with TNF-α (50 ng/ml) for different time periods. (E) Immunohistochemical staining of HMGB1 was conducted to evaluate the protein expression levels in human IVD tissue (scale bar: 100 μm). Western blot (F) and subsequent densitometric analyses (G) were performed to examine the effect of GL on the protein expressions of COL2A and ACAN. Western blot (H) and subsequent densitometric analyses (I) were used to examine the effect of GL on the protein expressions of MMP3 and ADAMTS5. All experiments were repeated three times, and data are presented as the mean±SD. * P<0.05.

HMGB1 is reportedly closely related to cholesterol metabolism [ 31, 34] and can be regulated by rosuvastatin in the cardiovascular system [ 35, 36] . Whether the protective effects of rosuvastatin in NP cells are mediated by HMGB1 remains unknown. Therefore, we first confirmed that HMGB1 expression was elevated in NP cells treated with TNF-α at different concentrations for 72 h and a constant concentration of 50 ng/mL for different stimulation time ( Figure 3A–D). Upregulation of HMGB1 was also observed in degenerative IVD tissues ( Figure 3E). To reveal the function of HMGB1 in TNF-α-treated NP cells, the HMGB1 inhibitor glycyrrhizinate (GL; Sigma Aldrich) was used. As shown in Figure 3F,G, in the presence of TNF-α, the protein expressions of ACAN and COL2A were decreased to 75% and 57% of those in the control group, yet it was increased to 121% and 95% by GL. On the other hand, GL administration attenuated the increase in the protein expressions of ADAMTS5 and MMP3 induced by TNF-α ( Figure 3H,I).

### HMGB1 inhibition or knockdown suppresses TNF-α-induced pyroptosis and senescence in NP cells

**Figure 4:**
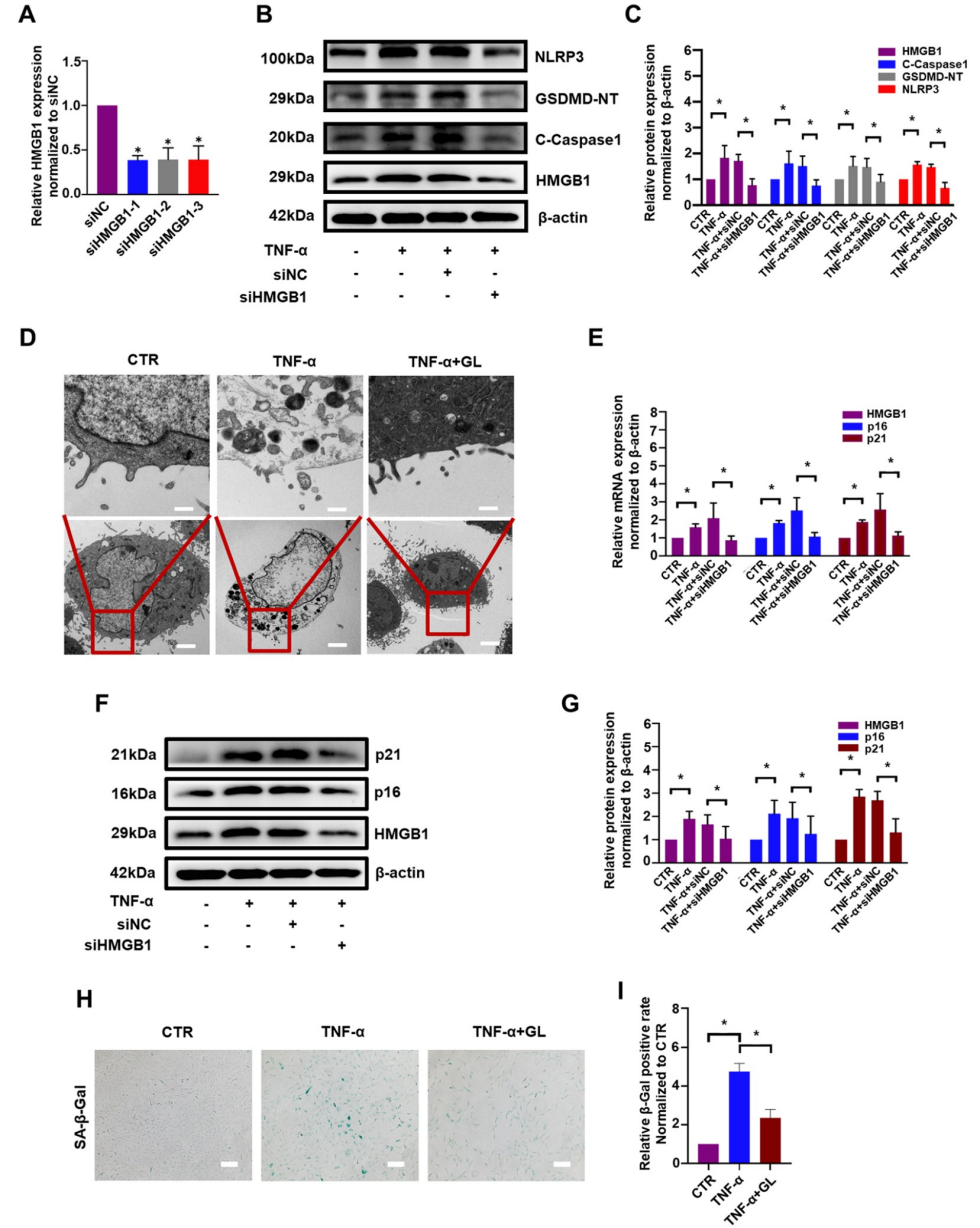
Figure 4 HMGB1 inhibition or knockdown suppressed pyroptosis and senescence in NP cells (A) Knockdown efficiency of the HMGB1 gene with different siRNA sequences. Western blot (B) and subsequent densitometric analyses (C) of pyroptosis-related proteins after HMGB1 knockdown. (D) TEM showed that the HMGB1 inhibitor maintained the membrane integrity of NP cells (scale bar for upper panel: 500 nm, lower panel: 5 μm). (E) Quantitative real-time PCR was used to detect the effect of HMGB1 knockdown on p21 and p16 mRNA levels. Western blot (F) and subsequent densitometric analyses (G) of the expressions of p21 and p16 after HMGB1 knockdown. Representative micrographs (H) and quantitative analysis (I) of NP cells stained for SA-β-gal in the different treatment groups (scale bar: 10 μm). All experiments were repeated three times, and data are presented as the mean±SD. * P<0.05.

To further validate the function of HMGB1, HMGB1 siRNA was introduced. According to the knockdown efficacy ( Figure 4A), si-HMGB1-1 was chosen for subsequent experiments. As shown in Figure 4B, western blot analysis demonstrated that the expressions of pyroptosis-related proteins, including NLRP3, GSDMD-NT, and cleaved caspase-1, were increased by TNF-α, but this change was markedly reversed by *HMGB1* knockdown; the corresponding densitometric analysis revealed that NLRP3, GSDMD-NT, cleaved caspase-1 and HMGB1 protein expressions were significantly enhanced (by more than 1.5-fold) by TNF-α. However, after *HMGB1* was knocked down to 45%, the levels of NLRP3, GSDMD-NT and cleaved caspase-1 were correspondingly decreased to 45%, 61% and 50%, respectively, compared with those in the knockdown control group ( Figure 4C). Furthermore, TEM revealed membrane rupture and pyroptotic vesicles in NP cells treated with TNF-α, whereas GL maintained the membrane integrity of NP cells in the presence of TNF-α ( Figure 4D). In addition, when HMGB1 mRNA expression was downregulated to 41%, the mRNA levels of p21 and p16 were markedly decreased to 44% and 42%, respectively, compared with those in the knockdown control group in response to TNF-α stimulation ( Figure 4E). Accordingly, western blot and corresponding densitometric analysis demonstrated that after *HMGB1* was knocked down to 63%, the protein levels of p21 and p16 were correspondingly reduced to 49% and 65%, respectively ( Figure 4F,G). Moreover, as verified by SA-β-gal staining and quantitative analysis, fewer positive cells were detected in the TNF-α and HMGB1 inhibitor groups than in the TNF-α group, which indicated that the HMGB1 inhibitor mitigated NP cell senescence ( Figure 4H,I).

### HMGB1 mediates the protective effects of rosuvastatin on NP cells stimulated by TNF-α

**Figure 5:**
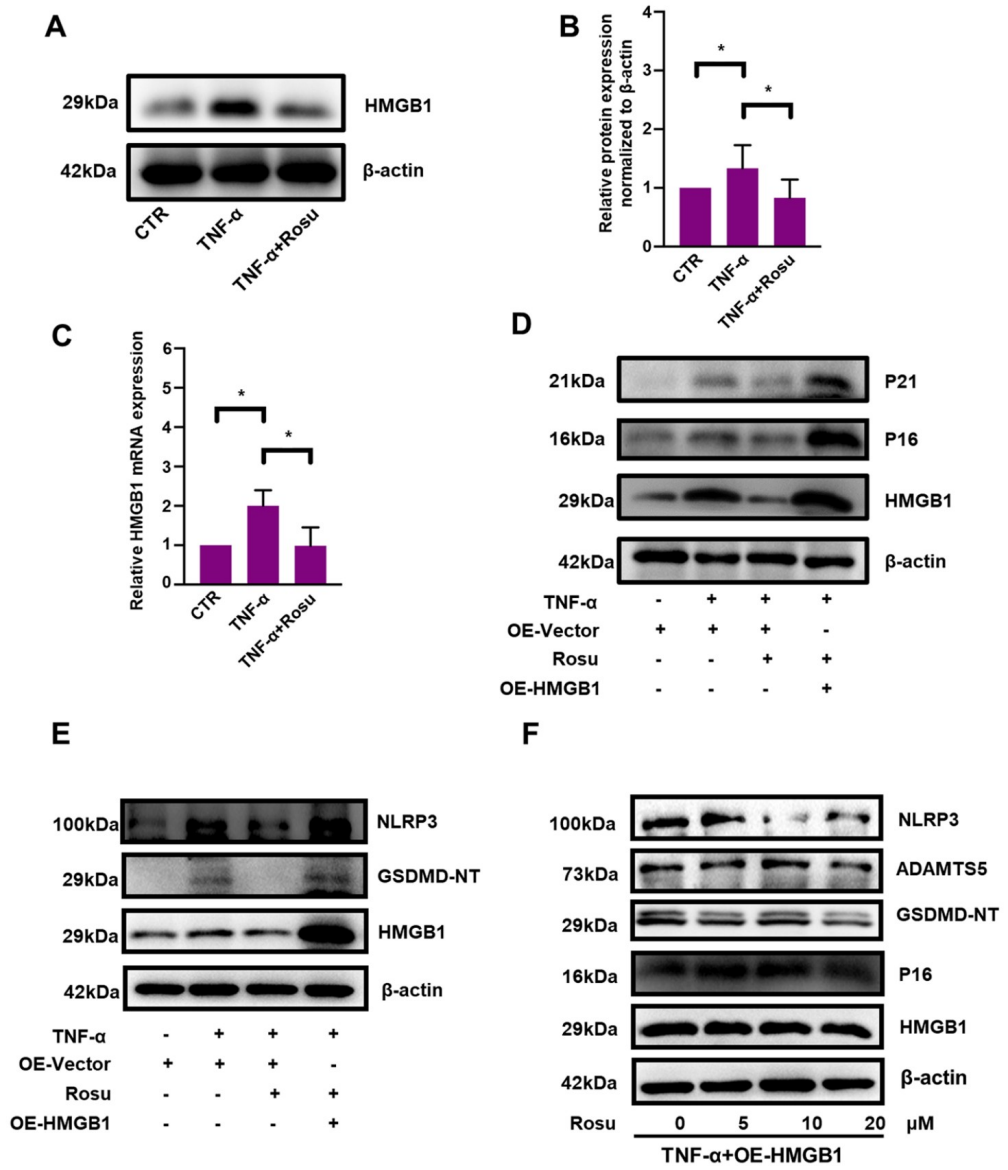
Figure 5 HMGB1 mediated the protective effects of rosuvastatin on TNF-α-stimulated NP cells Western blot (A) and subsequent densitometric analyses (B) were conducted to investigate the effect of rosuvastatin on HMGB1 expression. (C) The effect of rosuvastatin on HMGB1 expression was also explored by quantitative real-time PCR. Western blot analysis demonstrated that HMGB1 overexpression abrogated the protective effects of rosuvastatin on the senescence-related proteins p16 and p21 (D) and the pyroptosis-related proteins GSDMD-NT and NLRP3 (E). (F) Western blot analysis was conducted to investigate the protein expressions of HMGB1, p16, GSDMD-NT, ADAMTS5, and NLRP3 after treatment with TNF-α, HMGB1 plasmid and different concentrations of rosuvastatin. All experiments were repeated three times, and data are presented as the mean±SD. * P<0.05.

Subsequently, our western blot analysis results demonstrated that the level of HMGB1 was increased to 133% relative to the control level but was significantly decreased to 83% by rosuvastatin ( Figure 5A,B). In addition, although the mRNA level of HMGB1 was prominently increased by 2-fold in response to TNF-α, it was reduced to almost the control level by rosuvastatin ( Figure 5C). To further validate that the protective effect of rosuvastatin is achieved by downregulating HMGB1, we constructed a plasmid for HMGB1 overexpression. Rosuvastatin significantly alleviated the senescence and pyroptosis of NP cells, but the protective effects were abrogated by HMGB1 overexpression ( Figure 5D,E). To determine whether the detrimental effect of HMGB1 is affected by different doses of rosuvastatin, NP cells were treated with HMGB1 plasmid, TNF-α and different concentrations of rosuvastatin. As shown in Figure 5F, rosuvastatin suppressed p16, NLRP3 and AMAMTS5 protein expressions, but the changes were not statistically significant. In our study, HMGB1 was found to be a downstream molecule of rosuvastatin; therefore, it is reasonable to conclude that rosuvastatin does not alter the effect of HMGB1 overexpression.

### Rosuvastatin acts on NP cells via the NF-κB signaling pathway

**Figure 6:**
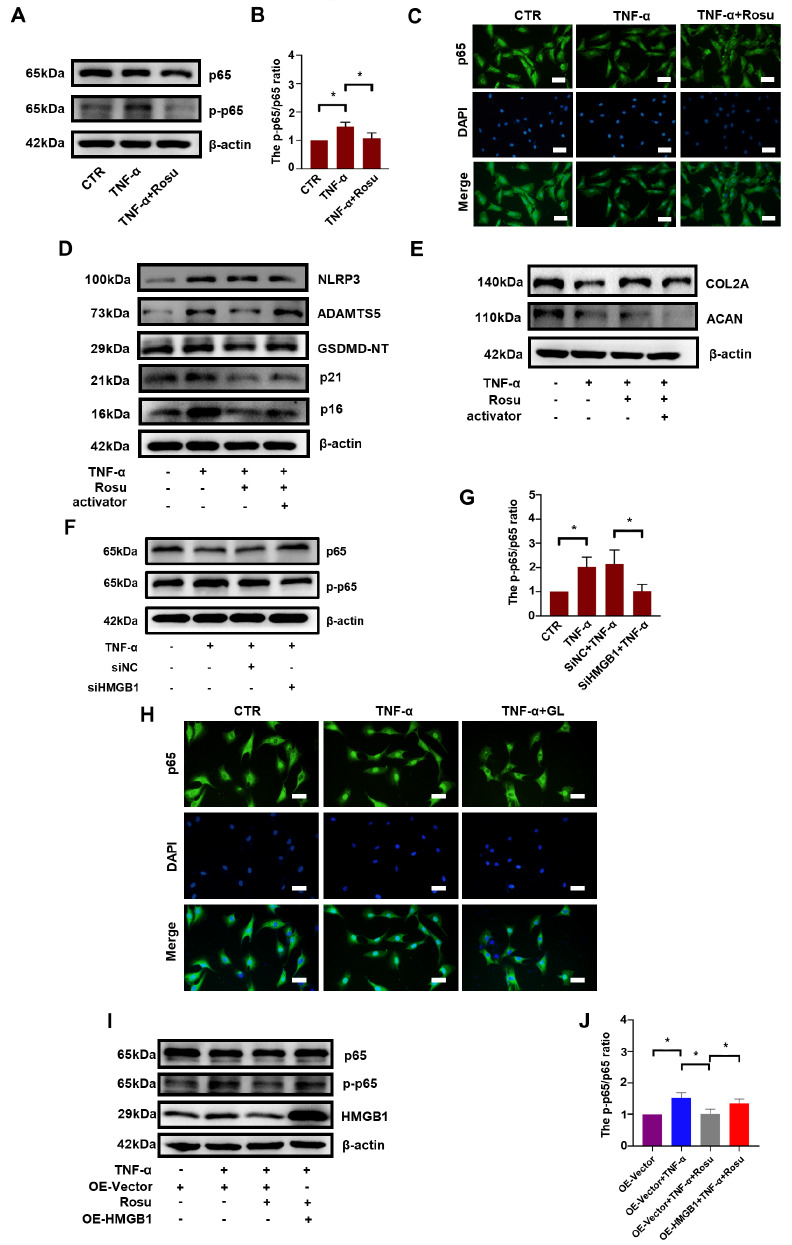
Figure 6 Rosuvastatin acts on NP cells via the NF-κB signaling pathway Detection of p65 phosphorylation by western blot analysis (A) and calculation of the p-p65/p65 ratio (B) after treatment with rosuvastatin. (C) The nuclear translocation of p65 after treatment with rosuvastatin was detected by immunofluorescence microscopy (scale bar: 40 μm). (D,E) The effect of NF-κB activator 1 on ECM degradation, senescence and pyroptosis was detected by western blot analysis in the presence of TNF-α and rosuvastatin. Western blot (F) and subsequent densitometric analyses (G) of the effect of HMGB1 knockdown on p65 phosphorylation. (H) The nuclear translocation of p65 after treatment with GL was detected by immunofluorescence microscopy (scale bar: 40 μm). Western blot (I) and subsequent densitometric analyses (J) were used to examine the phosphorylation state of p65 after HMGB1 overexpression. All experiments were repeated three times, and data are presented as the mean±SD. * P<0.05.

The NF-κB/p65 signaling pathway is involved in cell pyroptosis and senescence [ 37, 38] . Whether rosuvastatin regulates NF-κB/p65 in NP cells remains unknown. As shown in Figure 6A,B, TNF-α increased the level of phosphorylated p65 (p-p65), a key factor in the NF-κB pathway, by approximately 1.5-fold, but this effect was reduced by the administration with rosuvastatin. The nuclear translocation of p65 was also inhibited by rosuvastatin ( Figure 6C). To further clarify that the protective effect of rosuvastatin is achieved via the NF-κB/p65 pathway, an agonist named NF-κB activator 1 was applied. As shown in Figure 6D,E, the protective effect of rosuvastatin on ECM degradation, senescence and pyroptosis was abrogated by NF-κB activator 1. We then detected the phosphorylation state of p65 after *HMGB1* knockdown. Western blot analysis demonstrated that the p-p65/p65 ratio was significantly increased by TNF-α to approximately 200% but was decreased by 50% with *HMGB1* knockdown ( Figure 6F,G). Immunofluorescence staining also revealed that TNF-α promoted the nuclear translocation of p65, but this effect was inhibited by GL ( Figure 6H). To validate that HMGB1 is involved in this regulation, an HMGB1overexpression plasmid was applied. As shown in Figure 6I,J, HMGB1 overexpression significantly promoted p65 phosphorylation in the presence of TNF-α and rosuvastatin.

### Rosuvastatin alleviates IDD progression in a rat model

**Figure 7:**
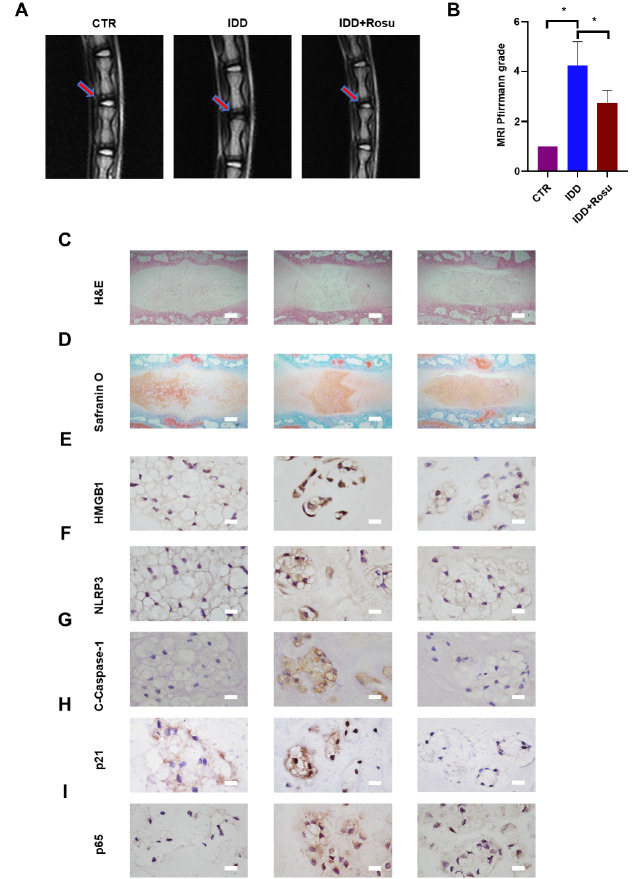
Figure 7 Rosuvastatin alleviated IDD progression in a rat model Representative MRI (A) and Pfirrmann grade analysis (B) of rat caudal discs 4 weeks after needle puncture. HE staining (C) and safranin O/fast green staining (D) of IVDs in the indicated groups 4 weeks after needle puncture. Scale bar: 1 μm. Immunohistochemical staining of HMGB1 (E), NLRP3 (F), c-Caspase-1 (G), p21 (H) and p65 (I) was conducted to evaluate the expression levels of these proteins in rat IVDs (scale bar: 100 μm). All experiments were repeated three times. * P<0.05.

To further verify the therapeutic potential of rosuvastatin, an IDD rat model was established by caudal IVD puncture. As shown in Figure 7A,B, sagittal lumbar MRI images showed decreased intensity in the rat tail IVD in the IDD group, and this effect was reversed by rosuvastatin. Moreover, HE and safranin O/fast green staining showed decreased levels of gelatinous NP tissue and disorganized annulus fibrosus in the IDD group, and this effect was significantly attenuated by rosuvastatin ( Figure 7C,D). Furthermore, the immunohistochemistry results revealed that the expressions of HMGB1, p65, the pyroptotic proteins NLRP3 and cleaved caspase-1, and the senescence-related protein p21 were increased in the IDD group and markedly decreased in the rosuvastatin group compared with those in the IDD group ( Figure 7E‒I).

## Discussion

IDD is a complex process that is resulted from multiple pathophysiological processes [7]. Enhanced catabolism and weakened anabolism in the ECM are characteristics of IDD [39]. Notably, our previous study showed that cholesterol level was elevated in NP cells treated with inflammatory cytokines [13], suggesting that cholesterol dysregulation may be another indicator of IDD. As an effective cholesterol modulator, rosuvastatin has been proven to regulate HMGB1 in cholestatic liver injury [40] and myocardial ischemia [ 35, 36] , but its function in IDD has not yet been fully explored. Because HMGB1 is involved in both cholesterol metabolism [31] and the inflammatory response [41], we hypothesized that rosuvastatin could serve as an anti-inflammatory agent by regulating HMGB1 in IDD. As expected, our study revealed that rosuvastatin suppressed HMGB1 expression while alleviating the detrimental effects caused by HMGB1 upregulation.

As reported previously, statins downregulate MMP3 and ADAMTS5 expressions in chondrosarcoma cells [42], and our previous studies demonstrated that atorvastatin regulated matrix metabolism in NP cells [22]. In the present study, *in vivo* experiments verified that rosuvastatin could protect IVDs from degeneration in rat models, and further experiments showed that the induction of the catabolic genes MMP3 and ADAMTS5 by TNF-α was suppressed by rosuvastatin. These findings indicate that rosuvastatin can restore the balance of ECM metabolism during IDD.

NP cell senescence is promoted as IDD progresses [43]. In addition, previous studies have proven that inhibiting NP cell senescence could alleviate IDD progression [ 44– 46] . Herein, Western blot analysis and β-Gal staining showed that rosuvastatin could alleviate senescence induced by TNF-α, indicating that rosuvastatin could protect NP cells from senescence in an inflammatory environment.

As reported previously, the inhibition of pyroptosis could be a therapeutic strategy for IDD [ 47, 48] , and rosuvastatin could regulate the NLRP3 inflammasome in the cardiovascular system [ 35, 36] , digestive system [49] and nervous system [50]. However, whether rosuvastatin can mediate pyroptosis in NP cells has not yet been proven. In this study, we verified that rosuvastatin could regulate NLRP3, cleaved caspase-1 and GSDMD expressions in the presence of TNF-α, as revealed by western blot analysis. TEM further showed the formation of membrane pores after TNF-α stimulation, while rosuvastatin inhibited pore formation and restored membrane integrity. These results indicated that rosuvastatin could suppress NLRP3 inflammasome activation and further inhibit pyroptosis in NP cells and that the inhibition of pyroptosis by rosuvastatin under inflammatory conditions might be a new strategy for treating IDD.

HMGB1 is involved in several cellular processes, including inflammation, tumor progression and cell differentiation [ 51, 52] . In contrast, HMGB1 upregulation could promote the expressions of cytokines such as TNF-α and IL-6 [53]. In NP cells, HMGB1 is upregulated and translocates after inflammatory stimulation, and HMGB1 can aggravate IDD by promoting apoptosis or suppressing autophagy [54]. Our western blot analysis, β-Gal staining and TEM results further showed the suppressive effects of HMGB1 inhibition on senescence and pyroptosis in NP cells in the presence of TNF-α. These findings suggest that HMGB1 is involved in IDD and could be a target of rosuvastatin.

The NF-κB pathway participates in several biological processes, including the inflammatory response, programmed cell death, and cell differentiation [55]. Aberrant activation of the NF-κB pathway could promote IDD progression [ 56, 57] . Rosuvastatin can regulate the activation of the NF-κB pathway in response to multiple stimuli [58]. HMGB1 can activate the NF-κB pathway by promoting cytokine production or directly binding to NF-κB subunits and potentiating the transcriptional activation of NF-κB [59]. The present study showed that phosphorylation of p65 was attenuated by rosuvastatin and HMGB1 inhibition or knockdown, while HMGB1 overexpression could promote p65 phosphorylation in the presence of TNF-α and rosuvastatin.

**Figure 8:**
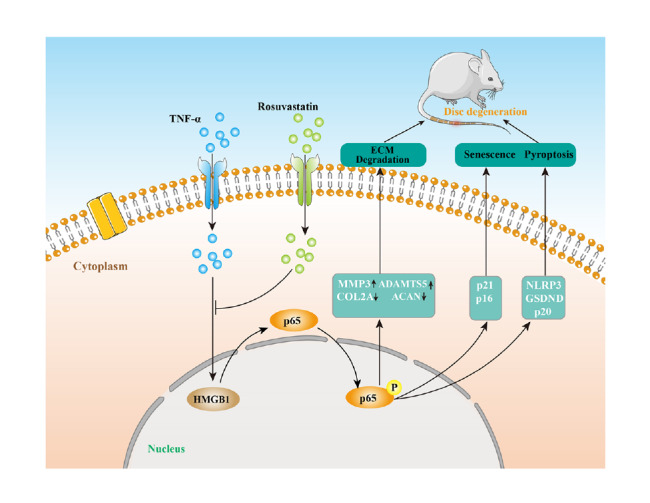
Figure 8 Schematic diagram of the effect of rosuvastatin on IDD Rosuvastatin suppresses TNF-α-induced matrix catabolism, pyroptosis and senescence by inhibiting the HMGB1/NF-κB signaling pathway in NP cells.

In conclusion, the current study demonstrated that rosuvastatin, a type of statin that is widely used in the clinic, protects IVDs from degeneration by alleviating TNF-α-induced matrix degradation and suppressing cell senescence and pyroptosis through the HMGB1-NF-κB signaling pathway ( Figure 8). Rosuvastatin may exert therapeutic effects to alleviate IDD.
